# Expanding access to pain care for frail, older people in primary care: a cross-sectional study

**DOI:** 10.1186/s12912-016-0147-5

**Published:** 2016-04-23

**Authors:** M. E. Muntinga, A. P. D. Jansen, F. G. Schellevis, G. Nijpels

**Affiliations:** Department of General Practice and Elderly Care Medicine, EMGO+ Institute for Health and Care Research, VU University Medical Center, Amsterdam, The Netherlands; NIVEL (Netherlands Institute for Health Services Research), Utrecht, The Netherlands

**Keywords:** Frail older people, Practice nurse, Pain, Comprehensive geriatric assessment, Access to health care, Primary care

## Abstract

**Background:**

Although untreated pain has a negative impact on quality of life and health outcomes, research has shown that older people do not always have access to adequate pain care. Practice nurse-led, comprehensive geriatric assessments (CGAs) may increase access to tailored pain care for frail, older people who live at home. To explore this, we investigated whether new pain cases were identified by practice nurses during CGAs administered as part of an intervention with the Geriatric Care Model, a comprehensive care model based on the Chronic Care Model, and whether the intervention led to tailored pain action plans in care plans of frail, older people.

**Methods:**

We used cross-sectional data from the older Adults: Care in Transition (ACT) study, a 2-year clinical trial carried out in two regions of the Netherlands. Practice nurses proactively visited older people at home and administered an in-home CGA that included an assessment of pain. Pain care-related agreements and actions (pain action plans) based on CGA results were described in a tailored care plan. We analyzed care plans of 781 older people who received a first-time CGA by a practice nurse for the presence of pain, pain location and cause, new pain cases, and pain action plans. We used descriptive statistics to analyze our data.

**Results:**

We found that 315 (40.3 %) older people experienced any type of pain. Practice nurses identified 20 (10.6 %) new pain cases, and 188 (59.7 %) older people with pain formulated at least one therapeutic or non-therapeutic pain action plan together with a practice nurse. More than half of the older people whose pain had already been identified by a primary care physician wanted a pain action plan. Most pain action plans consisted of actions or agreements related to continuity of care.

**Discussion and conclusion:**

Practice nurses in primary care can contribute to expanding older people's access to tailored pain care. Future researchers should continue to direct their focus at ways to overcome the barriers that restrict older people’s access to pain care.

## Background

Due to a changing health care system and the desire to age in place (the desire to remain living at home as long as possible [1, 2]), care for older people is rapidly shifting from an institutional to a primary care setting. As a consequence, primary care professionals are increasingly confronted with patients who suffer from degenerative diseases and multiple chronic conditions. Such complex health problems frequently go together with pain [3, 4]. It has been estimated that between 25 and 50 % of older people who live at home regularly experience pain [5]. Despite these numbers, research evidence suggests that barriers at a professional, patient and health system level limit older people’s access to adequate pain care services [6–8]. For instance, health care professionals may hold misconceptions and inherent bias, or may lack the knowledge or experience to adequately evaluate and treat pain in older patients [9–12]. In addition, older people themselves may avoid reporting their pain to health care professionals, use vague and varying terms to describe their complaints, or are reluctant to take medications [7, 13–16]. Health systems may pose practical constraints to adequate pain care, such as restricted access to services or financial burdens [8, 14]. As a result of these barriers, older people’s pain remains often undertreated or not recognized by health professionals [10, 17–19]. When undermanaged or unmanaged, pain can cause adverse health outcomes such as depression, anxiety, cognitive impairment and social isolation, which negatively impacts on older people’s health and social wellbeing, and, consequentially, increases the burden on health care systems [4, 10, 18, 20–23]. It is therefore essential that health professionals in primary care expand access to pain care by recognizing pain problems at an early stage and by providing pain care that is tailored to the older person’s individual need.

In response to the growing pressure on health care systems and the subsequent strain on elderly care services, governments, policy makers and researchers have been testing and implementing a type of care model characterized as comprehensive. Comprehensive care models provide a framework for the organization and delivery of integrated, patient-centered care, often in a primary or chronic care setting [24]. One of such models is the Geriatric Care Model [25], a comprehensive primary care model for frail, older people in the Netherlands based on the Chronic Care Model [26]. An important premise of the Geriatric Care Model is that, whenever feasible, treatment choices and decision making processes are guided by older people’s own needs and preferences. Within this premise, the Geriatric Care Model has three main objectives: to identify older people’s health and care needs at a timely stage, to enhance coordination between professionals at an individual and regional level, and to encourage older people’s involvement in their own care process. Central to the Geriatric Care Model is a proactive home visit program with comprehensive geriatric assessments (CGAs) administered by practice nurses [25]. In the Netherlands, practice nurses support primary care physicians in providing medical care for patients with chronic conditions, and mainly carry out organizational and guideline-based activities [27]. A CGA is a multidimensional evaluation that determines an older person’s medical, psychosocial, functional and environmental resources [25]. Together with, for instance, cognitive and functional status, pain is considered a major CGA domain, and is often assessed by means of unidimensional pain scales (such as the widely-used numerical rating scale (NRS)) or multidimensional pain scales [28]. Within the Geriatric Care Model, practice nurses use GCA results to further explore an older person’s pain care needs, and to present possible management and treatment options. During this process, practice nurses aim to actively involve older people in the decision making process, encouraging them to make a decision that best fits their needs. Pain care-related decisions are reported in tailored pain care plans.

The Geriatric Care Model may expand access to pain care for frail, older people in primary care in several ways. First, research suggests that expanding the primary care workforce to include health professionals that are not physicians (e.g. nurses) can maintain access to care, and that that nurse supplementation in primary care generally improved the quality of care delivery [29, 30]. Second, by proactively exploring pain complaints through administering a CGA, practice nurses may indentify pain and pain care needs at a more timely stage. Third, CGAs ensure that all aspects of an older person’s health and wellbeing are asked about and specified, and are often mentioned as an essential method for planning pain management strategies that are tailored to older people’s needs, preferences and environment [16, 31–35]. Finally, visiting older people in their home environment can provide practice nurses with important information about an older person’s context and lifestyle, and helps overcome logistical barriers to care [35]. The home setting can also reduce the power imbalance intrinsic to a client-nurse interaction in an institutionalized environment by increasing people’s agency [36, 37], which allows for a more collaborative relationship between caregiver and receiver. A collaborative relationship facilitates the recognition and targeting of pain management needs and goals, and encourages adherence to pain management [38, 39].

To understand whether in-home, practice nurse-led CGAs have the potential to overcome barriers to pain relief and expand access to tailored pain care for frail, older people, it is essential to gain insight in how often older people who received a CGA reported pain complaints, and to learn more about the pain care-related decisions made by older people with pain. We therefore used data from a trial that evaluated the effectiveness, cost-effectiveness and implementation process of the Geriatric Care Model to investigate whether new pain cases were identified by practice nurses during proactive, in-home CGAs, and to explore which pain management decisions were made as a result of these CGAs. By reporting our results, we aim to contribute to a better understanding of ways in which access to tailored pain care for frail, older people can be expanded.

## Methods

### Study design and participants

We used cross-sectional, first-time assessment data from the older Adults: Care in Transition (ACT) study, a 2-year clinical trial that implemented the geriatric care model among 1147 patients of 65 years and older from 35 primary care practices in the Netherlands [25]. Since the cluster assigned to the primary care practice designated the starting time of the intervention, older people participating in the ACT study received their first assessment at different times during the trial period. We included all ACT participants who received at least one home visit and assessment (*N* = 869). Due to the exclusion of 82 individuals with missing or incomplete first-time assessment data, our final study sample consisted of 781 older people. ACT participants were recruited in three steps. First, primary physicians selected patients based on a polypharmacy criterion (five or more drugs prescribed in the last three months) [40]. Subsequently, primary care physicians included all other patients who met a multidimensional definition of frailty (i.e., a loss of resources and a reduced reserve capacity for dealing with stressors in multiple domains of human functioning, such as the physical, psychological and social domain [41]). Exclusion was based on the following criteria: residence outside area of practice registration; residence in a nursing home; cognitive impairment or impaired mental status; critical or terminal illness [25]. Finally, eligible individuals were then contacted by telephone and asked to consider study participation. Final eligibility was established with the Program on Research for Integrating Services for the Maintenance of Autonomy case-finding tool for disability (PRISMA-7) [42]. Individuals with a PRISMA-7 score 3 or higher were considered frail, and invited to participate in the study [40].

### The geriatric care model

The Geriatric Care Model was implemented in two regions in the Netherlands between 2010 and 2014. The model aims to enable productive interactions between activated, informed patients and a proactive, prepared care team. Care teams consist of practice nurses, a geriatric expert team (a geriatric nurse and an elderly care physician), a family physician, a pharmacist, and care professionals involved in an older person’s care process. Practice nurses played a central role in the implementation of the Geriatric Care Model: they carried out in-home CGA’s and performed coordinating tasks such as arranging care and support services and organizing multidisciplinary team consultations (MTCs, that were attended by the practice nurse, the primary care physician, the geriatric expert team, a pharmacist and other relevant health care professionals) to discuss clients with complex health and care situations.

Every six months, older people received two proactive home visits from a practice nurse. During the first visit, the practice nurse carried out a CGA using the multidimensional web-based Community Health Assessment version 9.1 of the Resident Assessment Instrument (RAI-CHA) [43]. By means of a structured list of items, RAI-CHA users explore their client’s health problems and care needs. Pain-related items include items about frequency, intensity, duration and ability to control the pain. RAI-CHA items trigger Client Analysis Protocols (CAPs) in several domains (e.g. physical wellbeing, social functioning, living and safety). The CAPs help RAI-CHA users identify possible targets for care, and support care and service. When a CAP was triggered or a practice nurse observed a health problem or care need independently from the RAI instrument, she discussed possible management options with the primary care physician and drafted a tailored care plan. For each CGA result, practice nurses reported in the care plan whether the primary care physician had been aware of their patients’ problem or need or not. During the second visit, approximately two weeks after the first visit, the practice nurse addressed the assessment results with the older person. They explored the older person’s wishes regarding further care, informed and advised them about suitable care options, and stimulated their involvement in the decision making process. In case an older person desired a plan for their health problem or care need, a care goal and an action or agreement were formulated and recorded in a care plan. At all times, older people were given the opportunity to edit or remove care plan content. All participants consented to the use of the care plan for research purposes.

### Data collection

We used tailored care plans (*N* = 781) written by practice nurses and based on first-time, in-home CGA results related to pain (including practice nurses’ own observations) to report the following care plan outcomes: ‘prevalence of any type of pain’, ‘location and cause of pain’, ‘prevalence of new pain cases’ and ‘prevalence of new pain action plans’. We used baseline data from the ACT study to report health-related and sociodemographic characteristics of our study population [25]. ACT study baseline data were collected by trained project interviewers by means of computer-assisted interviewing. Health-related characteristics included quality of life, functional capacity and self-reported chronic diseases. Quality of life was measured with the 12-item Short Form questionnaire (SF-12), which measures quality of life using a mental component summary score (MCS) and a physical component summary score (PCS) [44]. Functional capacity was measured using the Katz-15 index of Independence in Activities of Daily Living (IADL) [45], and calculated using a sum score. Four major self-reported diseases (Diabetes Mellitus, depression, cancer and cerebrovascular disease) were assessed with The Older Persons and Informal Caregivers Survey Minimum DataSet (TOPICS-MDS) [46]. Sociodemographic characteristics included sex, age, living situation (independent alone, independent with others, home for the aged or residential care), and education (primary, secondary, higher).

### Data processing and analysis

Care plans were analyzed as follows: each care plan was read by two researchers who independently analyzed the care plan for the presence of pain (present, not present), new pain cases (present, not present, missing) and pain action plans (present, patient currently has adequate action plan, patient refuses action plan, reason action plan not present unknown). Care plans that mentioned pain as a CGA result were analyzed for the presence of a location and cause of the pain. If a care plan featured a pain action plan, the action plan was categorized as either a therapeutic intervention or a non-therapeutic intervention. *Prevalence of pain* was assessed by calculating the number of care plans that mentioned pain (both recently developed and persistent pain, and pain of different frequencies, patterns and intensities) as a result of a CGA (i.e. when the RAI instrument triggered a pain-CAP or a practice nurse identified pain independently from RAI). *Location and cause of pain* was assessed by categorizing practice nurses’ own care plan descriptions of pain location and cause, and by subsequently calculating the number of times each category was present in a care plan. ‘Location’ categories were as follows: back & neck, buttocks, joints, leg or legs, arm or arms, hand or hands, foot or feet, head, abdomen, respiratory tract, genitals, other. ‘Cause’ categories were as follows: arthritis of one or more joints, rheumatoid arthritis, polymyalgia, osteoporosis, peripheral arterial disease, persistent pain after recent trauma, persistent pain after past trauma, persistent pain after recent surgery, cancer pain, pain as a side effect of medication, cause pain unknown, other. *Prevalence of new pain cases* was assessed by calculating the number of care plans that mentioned that a pain complaint had not been identified by a primary care physician. *Prevalence of pain action plans* was assessed by calculating the number of care plans that contained both a pain care-related care goal and a pain care-related agreement or action. We distinguished the following two categories of pain action plans: (1) action plans that contained therapeutic interventions (pharmacological interventions and non-pharmacological interventions) and (2) action plans that contained non-therapeutic interventions (education-related interventions, continuity of care-related interventions or ongoing assessment-related interventions). The outcomes of the independent analyses were compared, and in case researchers disagreed, a final decision was reached by consensus.

We used descriptive statistics to analyse care plan data and ACT study baseline data. We performed independent T-tests and chi-square tests to compare health-related and sociodemographic characteristics of older people with and without any type of pain and with and without a pain care plan. Data were analyzed using SPSS Statistics version 20.

## Results

Care plan data show that 315 (40.3 %) of the 781 frail, older people who received a first time CGA reported any type of pain. Table 1 shows the characteristics of the 781 older people included in our sample, stratified for pain. The group with pain differed significantly from the group without pain: older people with pain were more often female, were younger, had lower scores on the physical component of the SF-12 questionnaire, more often reported IADL limitations and more often reported to suffer from osteoporosis, urinary incontinence and a depressed mood. Older people with pain most often experienced pain in their joints (38.7 %), neck and back (26.3 %) and legs (18.1 %). The cause of pain was most often arthritis (24.4 %) and persistent pain after past trauma (17.1 %). In 17.1 % of all pain complaints the cause of the pain was unknown.

**Table 1:** Characteristics of 781 frail, older people with and without any type of pain in the Netherlands who received an in-home, nurse-led comprehensive geriatric assessment

*N* = 781	Pain	No pain	*p*-value
	*N* = 315	*N* = 466	
Female (%)*	250 (79.4)	270 (57.9)	<0.001
Age*	79.0 SD 7.3 (64.9–97.1)	80.6 SD 7.4 (64.7–98.8)	<0.01
PCS*	30.5 SD 8.2 (11.0–53.6)	37.1 SD 9.3 (14.9–61.8)	<0.001
MCS	50.0 SD 10.3 (17.8–70.6)	50.3 SD 10.6 (13.9–70.5)	0.71
Katz IADL*	3.89 SD 2.7 (0–15)	3.45 SD 2.6 (0–13)	0.25
Living situation (%)			
Independent, alone	181 (57.5)	247 (53.0)	0.06
Independent, with others	106 (33.7)	191 (41.0)	
Home for the aged or residential care	28 (8.9)	28 (6.0)	
Education (%)			
Primary	124 (39.4)	142 (30.5)	0.09
Secondary	147 (46.7)	248 (53.2)	
Higher	43 (13.7)	74 (15.9)	
Self-reported conditions (%)			
Asthma, COPD	76 (24.1)	134 (28.8)	0.08
Diabetes Mellitus	101 (32.1)	152 (32.6)	0.43
Depression*	66 (21.0)	72 (15.5)	0.03
Cancer	36 (11.4)	52 (11.2)	0.48
Cerebrovascular disease	21 (6.7)	30 (6.4)	0.50
Osteoporosis*	123 (39.0)	121 (26.)	<0.001
Urinary incontinence*	125 (39.7)	157 (33.7)	0.05

*PCS* Physical Component Summary score of the Short Form-12 quality of life questionnaire, *MCS* Mental Component Summary score of the Short Form-12 questionnaire, *IADL* Independence in Activities of Daily Living

*Significant difference (*p* < 0.05)

### Prevalence of new pain cases and pain action plans

We found that practice nurses identified a total of 20 (10.6 %) new pain cases during the home visits. In 231 (73.3 %) cases, pain had already been identified by a primary care physician. In addition, we found that care plans of 188 (59.7 %) older people with pain featured tailored pain action plans; 127 care plans did not contain a pain action plan because older people were already receiving adequate treatment for their complaints (48 %), because older people refused a plan (18.9 %) or for unknown reasons (33.1 %). We found no significant differences in demographic characteristics and self-reported conditions between the group with and without an action plan. The majority of older people whose pain had been identified by a physician wanted a pain action plan (57.1 %), as did most (90 %) older people whose pain had been identified by a practice nurse.

### Categories of pain action plans

Care plans of the 188 people who wanted a pain action plan featured a total of 252 actions and agreements. Often, care plans featured more than one plan: 59 care plans featured two, and nine care plans featured three pain action plans. We found that pain action plans mostly comprised non-therapeutic interventions related to continuity of care, education and ongoing assessment (63.1 % of all actions and agreements, see Table 2). The majority of actions and agreements were related to coordination of care (55.6 %): a large share of older people wanted to be referred to their primary care physician or to another health care professional (such as a rheumatologist or a orthopaedic nurse), or wanted a referral to an outpatient pain clinic; others agreed to the practice nurse discussing their complaints with their primary care physician or in an MTC. Several times, practice nurses provided older people with information (such as written educational material), advice or instructions, for instance with the aim to increase a client’s concordance to pain medicine or to inform a client about effective pain medication use. In some cases, agreements about pain care centered on ‘watchful waiting’, i.e. practice nurses agreed to monitor an older person’s situation, or older people agreed to consult their family physician upon worsening of their pain. Occasionally, a practice nurse planned to measure the Ankle Brachial pressure Index (ABI) to investigate a vascular cause of the pain.

**Table 2:** Prevalence and type of pain action plans in care plans

Type of pain action plan	Pain action plans (*N* = 252)
*Continuity of care* N (%)	
Referral	
Practice nurse refers older person to family physician	67 (26.6)
Practice nurse refers older person to other healthcare professional	22 (8.7)
Practice nurse refers older person to outpatient pain clinic	9 (3.6)
Coordination of care	
Practice nurse consults with family physician	8 (3.2)
Practice nurse organises MTC multidisciplinary consultation	6 (2.4)
Watchful waiting	
Practice nurse actively monitors pain	15 (6.0)
Client consults with family physician when pain deteriorates	13 (5.2)
*Education* N (%)	
Practice nurse provides information and advice about pain	16 (6.3)
*Ongoing assessment* N (%)	
Practice nurse measures ABI	3 (1.2)
*Pharmacological interventions* N (%)	
Pain medication is started or changed	50 (19.8)
*Nonpharmacological interventions* N (%)	
Physiotherapy	14 (5.6)
Exercise	6 (2.4)
Occupational therapy, manual therapy	4 (1.6)
TENS, support material	5 (2.0)

*ABI* Ankle-Brachial pressure Index *TENS* Transcutaneous Electrical Nerve Stimulation *MTC* Multidisciplinary Team Consultation

Almost one-third (31.3 %) of pain action plans involved therapeutic interventions. The majority of these were pharmacological interventions that revolved around starting pain medication or changing the dose or frequency of existing pain medication. Non-pharmacological interventions were aimed at supporting daily functioning and reduce pain, for instance by planning physiotherapy and occupational therapy or increase daily exercise (e.g. swimming). Occasionally, practice nurses advised older people to use support material such as braces and belts, or to try complementary treatment such as TENS (Transcutaneous Electrical Nerve Stimulation) (Table 2).

## Discussion

The aim of this study was to explore whether practice nurse-led CGAs could expand access to pain care for frail, older people who live at home. Results show that practice nurses identified new pain cases and formulated new tailored pain action plans together with older people (including older people whose pain had already been identified by a primary care physician). The majority of pain action plans involved actions and agreements related to continuity of care.

### Prevalence of pain

We found that the prevalence of any type of pain in our population was 40 %. Compared to other studies, this number is low; previous research has reported prevalences of any type of pain among older people who live at home around 70 % [19, 47]), which suggests that our finding might be an underestimation of the actual prevalence. This underestimation could be explained by the fact that frail, older people still experienced barriers to reporting their complaints to the practice nurse. Also, older people whose pain had already been adequately managed may not have always reported their pain. However, the fact that RAI-CHA items included a question about ability to control the pain and care plans contained information about adequate management and treatments suggests that practice nurses were able to overcome this barrier to reporting pain. Furthermore, the potential underestimation could be explained by the method of data collection. The care plans used to investigate the prevalence of any type of pain were written by practice nurses in practice, which may have influenced prevalence outcomes in several ways. First, the quality and completeness of pain registration in care plans varied between practice nurses, which challenged data analysis. Second, since a major objective of the Geriatric Care Model was optimal involvement of older people in their own care process, the final care plan content was determined by older people themselves. This potentially may have caused pain-related content to have been left out or removed.

### Pain action plans

The majority of pain action plans involved actions and agreements related to continuity of care. An explanation for the latter could be related to practice nurses’ tasks and activities within the Geriatric Care Model, which were for a large part related to care coordination. A large share of people’s pain complaints had already been identified by a primary care physician prior to the CGA. This may suggest that usual care in the Netherlands is often successful at the initial identification of pain complaints. However, the fact that older people still made pain action plans with a practice nurse when proactively approached for a CGA could imply that pain care was not always adequately tailored to older people’s needs at the time of the CGA. As older people’s pain care needs may change over time, previously suitable management or treatment strategies may become insufficient. For instance, our results show that 20 % of all actions and agreements centered on pharmacological interventions aimed at starting pain medication or at changing existing pain medication. Previous research supports our findings: Kemp et al. reported that older people found the pain management strategies they used only moderately helpful [48], and Sawyer at al. found that older people remained in pain even with medication [19]. Explanations for older people’s wishes to adjust their pain action plans may be related to barriers to reporting pain care needs. Makris et al. explored older people’s perspectives regarding care seeking for restricting back pain, and found that reasons for not seeking care were their belief that the pain was age-related and therefore inevitable, negative attitudes regarding medical interventions, and the perceived importance of their pain in relation to other comorbidities [49]. Considering the mean age of older people with pain in this study (79 years) and the presence of comorbities, similar belief-based barriers may have existed in our study population. This suggests that a proactive approach could contribute to overcoming barriers to care for older people who live at home, as it helps identify needs for pain care in a population that otherwise may not have consulted a health care professional with their complaints.

The circumstances of older people with pain are often complex. Since older people are prone to suffer from other limitations, such as depressive symptoms, loss of functioning, and low quality of life [50], their pain is seldom an isolated problem. It has therefore been recommended to take a more comprehensive approach to pain care for frail older people, one that seriously considers clients’ needs and preferences [51]. Such an approach should prioritize the optimisation of ADL functioning, and combine pharmacological treatment with nonpharmacological and complementary therapies, such as exercise, massage and mindfulness meditation [51–54]. These recommendations emphasize the need for an assessment that, in addition to pain, explores other domains of functioning (including the social and environmental domain) as well as older people’s personal background, value system and beliefs. A CGA administered by a practice nurse could therefore provide a primary care team with important insights to tailor pain care to the needs and circumstances of frail, older people. In addition, there is evidence that nurse involvement could lead to improved health outcomes for older people: research suggests that nurse supplementation in primary care could lead to improved health outcomes for clients if the treatments delivered by nurses are proven to be effective and nurses themselves added to the improved delivery of the treatments [30]. However, whether this approach can contribute to improved health outcomes has yet to be established.

### Study limitations

This study has several limitations. First, as mentioned above, using data from care plans written in practice to collect information about pain prevalence may have caused the prevalence of pain in our population to be an underestimation; in addition, it limited our ability to develop categories of pain action plans and distinguish between categories, as goals, actions and agreements were not always clearly formulated by practice nurses and no standardized method for reporting actions and agreements was used. Second, the care plans only provided insight in pain action plans that resulted from interactions between older people and practice nurses, whereas our data show that a large number of plans involved referrals to a physician. Lack of insight in pain action plans that resulted from interactions between older people and their physicians may have caused an underestimation of the actual number of pain action plans that eventually resulted from the CGA, or a underestimation of the prevalence of pharmacological interventions. Finally, whether client outcomes can be improved depends on the extent to which pain action plans are actually carried out. While older people are more likely to adopt care when care is tailored to their needs, our results do not provide insight in the extent to which pain action plans were put into practice. Literature suggests that the nature of the relationship between an older client and their caregiver plays an important role in the extent to which older people accept services and change their health behavior [55]. More research in this area could help increase our understanding of the processes that underlie older people’s decision making with regards to pain care.

## Conclusions

Our results suggest that the pain care needs of frail, older people who live at home are not always met. Proactive, practice nurse-led CGAs have the potential to reduce the number of unmet pain care needs by expanding access to tailored pain management in primary care. Regular follow up after initial identification of pain complaints and taking into account older people’s personal background, value system and beliefs can help nurses to further tailor pain care to individual needs and preferences. Since barriers to care seeking majorly restrict older people’s access to pain care, we recommend that efforts toward overcoming these barriers should be prioritized in future research and practice.

### Ethics and consent to participate

The ACT study was approved by the medical ethics committee of the VU University medical centre. All participants signed an informed consent form.

### Consent to publish

Not applicable.

### Availability of data and materials

All the data supporting the presented findings is contained within the manuscript.

## Abbreviations

ABI: Ankle Brachial pressure Index
ACT: older Adults: Care in Transition
CAP: Client Analysis Protocol
CGA: comprehensive geriatric assessment
IADL: Independence in Activities of Daily Living
MCS: mental component summary score
MTC: multidisciplinary team consultation
NRS: numerical rating scale
PCS: physical component summary score
PRISMA: Program on Research for Integrating Services for the Maintenance of Autonomy
RAI-CHA: Community Health Assessment version of the Resident Assessment Instrument
SF: short form
TENS: Transcutaneous Electrical Nerve Stimulation
TOPICS-MDS: The Older Persons and Informal Caregivers Survey Minimum DataSet
